# Skipping breakfast and excess weight among young people: the moderator role of moderate-to-vigorous physical activity

**DOI:** 10.1007/s00431-022-04503-x

**Published:** 2022-06-01

**Authors:** José Francisco López-Gil, Pedro Antonio Sánchez-Miguel, Miguel Ángel Tapia-Serrano, Antonio García-Hermoso

**Affiliations:** 1 Departamento de Expresión Plástica, Musical y Dinámica, Facultad de Educación, Universidad de Murcia (UM), 30100 Murcia Región of Murcia, Spain; 2 Health and Social Research Center, Universidad de Castilla-La Mancha (UCLM), 16071 Cuenca, Spain; 3 Departamento de Didáctica de la Expresión Musical, Plástica y Corporal, Grupo Análisis Comportamental de la Actividad Física y el Deporte (ACAFYDE), Facultad de Formación del Profesorado, Universidad de Extremadura, Av. de la Universidad, 10071 Cáceres, Spain; 4 Navarrabiomed, Hospital Universitario de Navarra (HUN), Universidad Pública de Navarra (UPNA), IdiSNA, 31008 Pamplona, Navarra, Spain

**Keywords:** Eating behavior, Lifestyle, Youths, Obesity, Fasting

## Abstract

The present study aimed to test whether the daily minutes of moderate-to-vigorous physical activity (MVPA) engaged moderate the relationship between breakfast status and excess weight (i.e., overweight and/or obesity) in a Spanish sample of young people. A cross-sectional study involving a total sample of 2890 Spanish schoolchildren (46% girls) aged 6−17 years (*M* = 12.3 ± 2.6) was conducted. To determine the habit of having breakfast, a dichotomous item about breakfast status (yes/no) from the Mediterranean Diet Quality Index for children and teenagers (KIDMED) was used. Physical Activity Questionnaire for Older Children and the Physical Activity Questionnaire for Adolescents were fulfilled to offer an estimation of the minutes of MVPA that individuals had in the last seven days. Body mass index (BMI) was converted into z-scores and, therefore, excess weight status (i.e., overweight and/or obesity) was established according to World Health Organization criteria for sex and age. Skipping breakfast was positively related with BMI (z-score) and excess weight. Moderation analyses suggested that daily MVPA minutes moderated the association between skipping breakfast and BMI (boys: *β* =  − 0.175; girls: *β* =  − 0.073) or the excess weight (boys: *OR* = 1.10; *CI* 95%, 1.02 to 1.07; girls: *OR* = 1.14; *CI* 95%, 1.04 to 1.24), meaning that physical activity of sufficient intensity seems to reduce the effect of skipping breakfast on the body weight status of young people.

*Conclusion*: Our results indicate that promotion of having breakfast should be accompanied by daily MVPA, as young participants who have breakfast and with higher daily MVPA seem to be more likely to have no excess weight.

**Table:** 

**What is Known:** *• Some of the well-studied factors associated with childhood obesity have been skipping breakfast and insufficient physical activity.* *• Some studies have pointed out the association between having breakfast and both body mass index and physical activity level, in isolation.*	
**What is New:** *• Daily minutes of moderate-to-vigorous physical activity moderate the association between skipping breakfast and excess weight.* *• Breakfast promotion as a healthy eating habit should be accompanied by increases in moderate-to-vigorous physical activity, since participants who have breakfast and with higher moderate-to-vigorous physical activity seem to be more likely to have no excess weight.*

**Supplementary information:**

The online version contains supplementary material available at 10.1007/s00431-022-04503-x.

## Introduction

Excess weight (understood as the sum of overweight and obesity) in young people is one of the most relevant concerns in global public health, although it arose as a serious matter some decades ago [1]. Thus, having excess weight during childhood and adolescence is related to detrimental health problems throughout the life-course [2, 3]. The least invasive and most widely used approach to treating obesity in childhood is lifestyle modification, including diet quality, physical activity (PA) levels and sedentary behaviors, and behavioral change techniques to help maintain positive changes and avoid relapses [4].

Skipping breakfast has been one of the well-studied factors associated with childhood obesity [5]. In this sense, breakfast has been pointed out as the most essential meal of the day [6]. Children and adolescents who skip breakfast could not be able to obtain the nutrients missed with the rest of meals of the day [7] and, as was previously indicated, an insufficient nutrient consumption may have long-term harmful consequences on their health [8]. Moreover, a systematic review with meta-analysis has suggested that eating breakfast daily can decrease the risk of childhood obesity by 34% [9]. Similarly, another systematic review has revealed that skipping breakfast may be a simple indicator of the risk of excess weight and metabolic diseases [10]. Despite this, a growing trend in skipping breakfast from childhood to adulthood has been described [11, 12], highlighting higher prevalence in girls than in boys [13, 14].

PA, understood as any bodily movement produced by skeletal muscles that requires energy expenditure, provides mounting benefits for health, especially when it is practiced at moderate-to-vigorous intensity [15]. Thus, moderate-to-vigorous PA (MVPA) refers to the PA that is performed at > three metabolic equivalents of task (METs) (i.e., > three times the intensity of rest) [15]. An insufficient level of MVPA has been recognized as one of the principal risk factors of excess weight [16]. In this regard, it has been also suggested that sufficient MVPA could reduce the risk of childhood obesity by 30% [9]. Based on the negative influence of sedentary behavior on health and the positive health effects of regular engagement in MVPA, the new World Health Organization (WHO) guidelines have been developed and updated and no longer point to the need for 60 min of MVPA every day (minimum) [17], but rather a daily average of at least 60 min in each week [18]. In relation to breakfast habits, a previous cross-sectional study including children from 12 different countries found that frequent breakfast consumption was associated with a higher proportion of time in both light PA and MVPA [19]. Similarly, a randomized crossover trial found that adolescents (girls) spent more time in PA before and after school when a standardized breakfast was consumed daily than when consumed intermittently across the week.

Obesity is a complex multifactorial condition with several possible environmental-, biological-, and behavioral-related factors [20]. Regarding behavioral factors, low levels of PA and inadequate dietary habits could lead to an imbalance between energy intake and energy use, which are consistent with an increased risk of having excess weight in an obesogenic environment (from an energy balance perspective) [21]. In this sense, some studies have been performed to determine the relationship between both dietary habits (e.g., breakfast habits) and PA and excess weight [9, 10]. For example, a meta-analysis by Poorolajal et al. [9] including 286,804 children and adolescents from 33 different countries found that eating breakfast every day and sufficient PA were individually associated with lower odds of having excess weight, among other lifestyle behaviors. Similarly, one longitudinal study among Brazilian adolescents showed that those who had breakfast frequently showed lower adiposity (regardless PA level), while trunk adiposity was lower in adolescents with increased PA level [22]. More specifically, some studies found that MVPA did not condition the association between skipping breakfast and excess weight [23, 24]. However, to date, literature investigating if the relationship between skipping breakfast and excess weight in young people is moderated by daily MVPA engaged is still scarce. Thus, identifying a moderator of an effect helps to establish the boundary conditions of that effect or the circumstances, stimuli, or type of people for which the association is large versus small, present versus absent, positive versus negative, and so forth [25]. Given the increases in the prevalence of excess weight and breakfast skippers, knowing whether daily MVPA moderates the relationship between breakfast and excess weight status could be relevant as a first step in establishing more specific recommendations and intervention programs.

Based on the above, our hypothesis is that skipping breakfast could increase the odds of having excess weight among children and adolescents [10] and daily MVPA level could decrease this association [9]. Consequently, this study attempts to verify whether the daily minutes of MVPA engaged moderate the relationship between skipping breakfast and excess weight in a sample of Spanish young people aged 6–17 years.

## Methods

### Population and study design

All primary and secondary schools in the Valle de Ricote (Region of Murcia, Spain) were asked to take part in this research. Similarly, participants from 22 secondary schools from the province of Cáceres (Extremadura, Spain) were enrolled. For this study, participants were recruited by a convenience sample. A final sample of 2890 Spanish schoolchildren (46% girls) aged 6–17 years were included. Of the total number of participants, 1318 (46%) were from Murcia and 1572 (54%) from Extremadura. Information about the selection of the study sample is shown in Fig. S1. Likewise, the characteristics and differences according to the participants’ exclusion/inclusion are shown in Table S1. Children, adolescents, their families, and school staff were previously informed about the study’s procedures. To be included in this study, an informed consent form signed by parents or legal guardians was required. Regarding inclusion criteria, we only included schoolchildren aged 6–17 years with informed consent signed by parents or legal guardians. In relation to exclusion criteria, schoolchildren did not enroll when they (a) did not participate in Physical Education lessons, since questionnaires and tests were performed during this subject; (b) had some kind of dysfunction that reduced the participation in PA (i.e., motor problem, any disease); and (c) were under some drug therapy.

The Ethics Committee of the University of Extremadura (ID Nº. 89/2016) and the Bioethics Committee of the University of Murcia (ID Nº. 2218/2018) approved the study protocols, and it was conducted in line with the Declaration of Helsinki and with full respect for human rights.

### Procedures

#### Breakfast status

To determine the habit of breakfast, a dichotomous item about breakfast status (yes/no) was used from the Mediterranean Diet Quality Index for children and teenagers (KIDMED) [26]. According to this question, children’s and adolescents’ breakfast was classified as breakfast or skipping breakfast. The questionnaires were administered and managed by trained staff.

#### Anthropometric measures

To measure the body height of participants, a portable height rod (precision: 0.1 cm) was used. Bodyweight was determined by an electronic scale (precision: 0.1 kg). Body mass index (BMI) was computed by dividing body weight (in kg) by body height (in meters squared), and, then, BMI was converted into z-scores and, therefore, excess weight status (i.e., overweight and/or obesity) was established according to World Health Organization criteria for sex and age [27, 28]. All measurements were performed by the same researcher in the Region of Murcia and another in Extremadura, respectively.

#### Moderate-to-vigorous physical activity

Participants fulfilled the Physical Activity Questionnaire for Adolescents (PAQ-A) or the Physical Activity Questionnaire for Older Children (PAQ-C) to provide an estimation of the time spent in MVPA during the last seven days [29, 30]. These tools have been previously translated and validated into Spanish and include nine items scored on a [31, 32].

Respecting individuals aged 6–7 years, former recommendations about the fulfillment of PAQ-C were adopted [33], as this tool was validated for youths from 8 to 14 years old. Thus, these recommendations indicate that parents can help their children to fulfill the questionnaire, without offering any guidelines for responding.

Furthermore, the mean of daily minutes of MVPA was computed following Saint-Maurice et al.’s equation [34] and used for further analysis.

#### Covariates

Participants’ sex and age were self-reported. The annual household income was considered as a proxy of the level of socioeconomic status (SES). Data on this matter was acquired by the Extremadura Statistics Institute and the Regional Statistics Center of Murcia, which categorize the socioeconomic status in relation to the annual household income in each of the municipalities of the Extremadura and the Region of Murcia, respectively. The choice of these covariates was based on the scientific literature [13, 14].

### Statistical analysis

The participants’ characteristics of the analyzed sample were shown as frequency distribution for categorical variables and as mean and standard deviation (SD) for continuous variables. The assumption of normality was checked by the Kolmogorov–Smirnov test. Differences between sexes were verified by Student’s *t* test. Binary logistic regression analyses, adjusted by potential covariates (age, region, and SES), were computed to verify the associations between daily minutes of MVPA, breakfast status, and excess weight status. Moderation analyses were conducted using PROCESS macro 4.0 in IBM SPSS software (IBM SPSS Statistics for Windows, Version 25.0, Armonk, NY, USA). The PROCESS macro applies ordinary least squares (OLS) analysis to estimate moderation models (model 1 in PROCESS) using BMI z-score/excess weight status as dependent variables, breakfast status as the independent variable, and daily MVPA as the moderator variable, with a bootstrapping-resampling procedure (10,000 samples) [35]. Simple linear regression analyses were performed to assess the relationship of the daily MVPA with breakfast status and BMI z-score. Furthermore, the pick-a-point approach was performed as a method for probing moderation, including arbitrary values (i.e., mean and ± 1 SD) for daily MVPA. These values were categorized into low (− 1 SD), medium (mean), and high (+ 1 SD). For simple linear regression analyses, we used the following parameters: effect size (*f* [2]) = 0.02, an alpha (α) error probability = 0.05, a statistical power (1−*β*) = 0.95, number of dependent variables = 1, and number of predictors = 5. Therefore, the minimum number of participants was 652. Logistic regression analyses were used to examine the relationship of the daily MVPA with breakfast status and excess weight status. For logistic regression analyses, we used the following formula *n* > 10 (*k* + 1) [36], where *k* is the number of independent variables. Therefore, as we had five independent variables, the minimum sample of participants required was 600.

## Results

Table 1 indicates the descriptive information of the study’s participants. The prevalence of excess weight was greater in boys (43%) than girls (35%). Conversely, the number of participants skipping breakfast was higher in girls (13%) than boys (9.2%). Significant differences were found between sexes for both excess weight status (*p* < 0.001) and breakfast status (*p* = 0.001). Moreover, the level of daily MVPA was higher in boys than in girls (*p* < 0.001).

**Table 1:** Participants’ characteristics of the analyzed sample according to sex (*N* = 2890)

**Variables**	**Boys (*n*** **= 1552; 54%)**		**Girls (*n*** **= 1338; 46%)**		***p***
	**Mean/*n***	**SD/%**	**Mean/*n***	**SD/%**	
Age (years)	12.2	2.7	12.5	2.8	0.001
Annual household income (€)	20,527.3	2421.7	20,507.2	2485.8	0.827
Region of Murcia (%)	691	45	627	59	0.208
Extremadura (%)	861	56	711	41	
Children (%)	660	43	466	35	0.253
Adolescents (%)	892	58	872	65	
Weight (kg)	50.7	17.0	48.3	13.8	< 0.001
Height (cm)	155.2	16.8	152.5	14.5	< 0.001
BMI (z-score)^a^	0.8	1.3	0.5	1.2	< 0.001
Excess weight status^a^ (%, yes)	660	43	466	35	< 0.001
Breakfast status (%, skipping)	143	9.2	174	13	0.001
PAQ-C/PAQ-A (score)	1.7	0.1	1.4	0.1	< 0.001
Daily MVPA mean (min)	7.1	3.1	5.7	3.0	< 0.001

*BMI* body mass index, *MVPA* moderate-to-vigorous physical activity, *PAQ-A* Physical Activity Questionnaire for Adolescents, *PAQ-C* Physical Activity Questionnaire for Older Children

^a^Excess weight established by the sum of participants with overweight or obesity according to the World Health Organization criteria [27, 28]

**Fig. 1:**
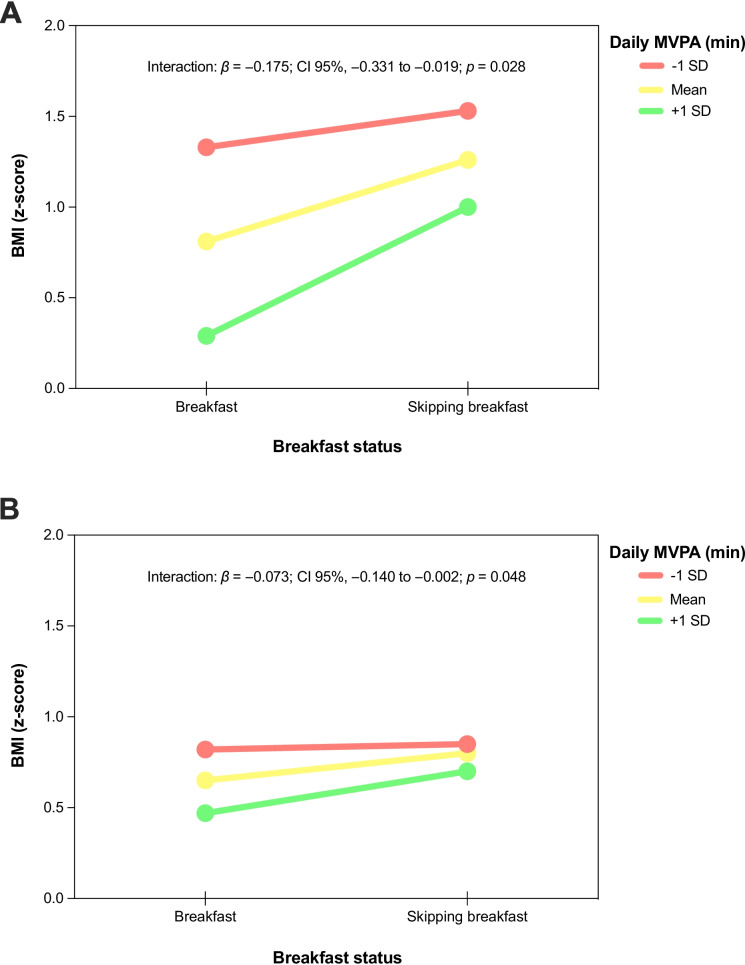
Moderator role of daily moderate-to-vigorous physical activity minutes in the association between breakfast status and body mass index (z-score). **A** Moderation model for boys. **B** Moderation model for girls. Adjusted by age, region, and socioeconomic status. BMI, body mass index; MVPA, moderate-to-vigorous physical activity

Figure 1 displays the moderator role of daily MVPA in the association between breakfast status and BMI (z-score). Daily MVPA minutes moderated the association between skipping breakfast and BMI (z-score) in both boys (*β* =  − 0.175; *CI* 95%, − 0.331 to − 0.019) and girls (*β* =  − 0.073; *CI* 95%, − 0.140 to − 0.002).

Table 2 displays the moderator role of MVPA level in the association between breakfast status and excess weight status. Skipping breakfast was associated with higher odds of having excess weight in boys (*OR* = 1.83; *CI* 95%, 1.18 to 2.86) and girls (*OR* = 1.98; *CI* 95%, 1.27 to 3.07). Daily MVPA skipping breakfast was inversely linked to excess weight in boys (*OR* = 0.55; *CI* 95%, 0.40 to 0.76) and girls (*OR* = 0.81; *CI* 95%, 0.65 to 0.99). Finally, daily MVPA minutes moderated the association between skipping breakfast and excess weight in boys (*OR* = 1.10; *CI* 95%, 1.02 to 1.07) and girls (*OR* = 1.14; *CI* 95%, 1.04 to 1.24).

**Table 2:** Association between daily minutes of moderate-to-vigorous physical activity and breakfast status and excess weight status

	**Excess weight status**^**b**^					
**Predictors**	***β***	**SE**	***p***	***OR***	**LLCI**	**ULCI**
	**Boys**					
Breakfast status^a^	0.607	0.227	0.008	1.83	1.18	2.86
Daily MVPA (min)	− 0.601	0.163	< 0.001	0.55	0.40	0.76
Breakfast status^a^ × daily MVPA (min)	0.091	0.034	0.008	1.10	1.02	1.17
	**Girls**					
Breakfast status^a^	0.681	0.225	0.002	1.98	1.27	3.07
Daily MVPA (min)	− 0.215	0.107	0.048	0.81	0.65	0.99
Breakfast status^a^ × daily MVPA (min)	0.127	0.046	0.006	1.14	1.04	1.24

*MVPA* moderate-to-vigorous physical activity. Adjusted by age, region, socioeconomic status, and quality diet

^a^Breakfast status coded as follows: 0–breakfast (reference group) and 1–skipping breakfast

^b^Excess weight status coded as follows: 0–no excess weight (reference group) and 1–excess weight

## Discussion

The present study suggested that the relationship between skipping breakfast and excess weight was moderated by daily minutes of MVPA in two Spanish regions. Although the number of breakfast skippers was higher among girls and the number of participants with excess weight was greater in boys, the moderator role of MVPA in the association between skipping breakfast and both BMI (z-score) and excess weight was consistent in both sexes. One possible reason is that girls who eat breakfast in a daily basis have shown a higher quality breakfast than boys [37]. Supporting this idea, different breakfast habits among European boys (i.e., higher intake of breakfast cereals, fruit, milk, and dairy, and fewer sugar-sweetened beverages) and girls (i.e., lower intake of pasta, rice, and others) have been inversely related to excess weight [38]. Despite its cross-sectional design, our results indicate that promotion of a healthy eating habit such as having breakfast should be accompanied by increases in daily MVPA, as young participants who have breakfast and with higher daily MVPA seem to be more likely to have no excess weight [38].

In accordance with the present results, previous studies have indicated that skipping breakfast is linked with obesity [5, 9, 10]. There are several factors which could justify this finding. First, breakfast skipping is related to changes in decreased satiety and higher appetite, which may result into overeating and altered insulin sensitivity [39]. Second, having breakfast is helpful for controlling appetite, and it can also enhance blood glucose levels and increase insulin sensitivity in the following meals [39]. Third, skipping breakfast can extend the overnight fast and more fasting time may result into increased release of ghrelin (a peptide hormone that promotes hunger) [40]. Fourth, children who skipped breakfast could have a low overall diet quality [41], which could lead to excess weight [21]. Fifth, cultural differences related to meal timing among countries could influence on excess weight [42]. For instance, Spain is located relatively westward within its time zone, resulting in sun rise and sun set occurring at a later time as compared to many other countries within the same time zone. The late meal timing in Spain is thus less extreme in comparison with solar time and it has been related to greater BMI and waist circumference in Spanish schoolchildren [43]. Notwithstanding the above, the mechanisms linking skipping breakfast and excess weight are still unclear [13].

Another important finding was that daily MVPA and excess weight were inversely associated. This fact is consistent with the scientific literature [44, 45]. Thus, higher levels of PA (including non-exercise activity thermogenesis) have been linked to lower BMI and body fat percentage, as well as for those who have excess weight, increased PA levels can be effective for reducing BMI [44, 45] and body fat percentage [45]. More specifically, MVPA has proven to be especially relevant in the prevention and treatment of childhood and adolescent obesity [44]. Notwithstanding, PA levels are mainly low among young people with obesity [46]. Moreover, a meta-analysis by Wang et al. [47] found that only the 31% of single PA had significant and favorable results to prevent childhood obesity. However, it is important to take into account that a significant body of research indicates that small changes in nutrition in addition to PA are also necessary to control weight [21].

Findings also seem to indicate that a sufficient amount of daily MVPA could moderated the association between skipping breakfast and excess weight in both sexes. Some studies have shown that MVPA was not a mediator variable in this relation neither in girls nor boys (adolescents), as well as in Latin and African American girls with excess weight [23, 24]. Conversely, Albertson et al. [48] revealed in a longitudinal study that PA is a mediator in relationship between eating breakfast and BMI over time in White and Black adolescent girls. Based on our findings, it seems that although young people who skip breakfast show higher risk of suffering obesity [9], greater levels of PA might be a valuable contributor to energy balance and weight control [45]. There are some mechanisms which could explain the associations obtained. As was previously mentioned, breakfast skipping is related to variations in decreased satiety and appetite, which may result into overeating and altered insulin sensitivity [39]. It might be explained by the reason that a higher PA level may lead to an increase in total calories consumed. Nevertheless, individuals with greater PA levels may be more sensitive to appetite control system by improved compensatory regulations for the density and energy content of food [49]. Supporting this idea, Schubert et al. [50] in their meta-analysis showed that exercise is successful to induce a caloric deficit (in a short term) and that people do not tend to compensate for the energy expenditure during physical exercise in the following hours post exercise by modifying food consumption. Additionally, another meta-analysis has shown that acute exercise might affect appetite by suppressing levels of some hormones (e.g., ghrelin), which could facilitate alterations in food/beverage consumption post-acute exercise. Also, a systematic review with meta-analysis conducted by García-Hermoso et al. [51] pointed out the efficacy of interventions including aerobic exercise on insulin resistance indicators in youths with obesity. In relation to the previously mentioned Spanish’s late meal timing, one study showed that a large dinner or late-evening snacking is associated with higher BMI only in children with low PA levels, suggesting that increased PA levels seem to compensate for the negative effects of late-night excessive caloric intake on children’s BMI [52]. Similarly, another study found that children at the lowest quartile of PA level had a positive association between the calories consumed at dinner and BMI, being this association inverse for children at the highest quartile of PA level [52]. These same authors found that high-energy intake at dinner and evening snack was related to skipping breakfast in children. However, the dinner consumption was not assessed in this study. Based on these facts, it is hypothesized that MVPA could moderate the association of skipping breakfast on excess weight in both boys and girls.

Breakfast is one of the more debated meals, contemplated as the most essential of the day on some occasions [6] and questioned in others [53]. For instance, a recent systematic review with meta-analysis concludes that breakfast consumption should be promoted to increase the macronutrient quality and food intake in both children and adolescents [54]. Conversely, one systematic review and meta-analysis including randomized controlled trials by Sievert et al. [55] (in adults) pointed out that caution is required to recommend breakfast for reducing body weight, since it may have the contrary result, regardless of established breakfast habit. Breakfast may not or may be the most essential meal, but it is undoubtedly a meal that needs further research [56]. What our results do suggest to point to is that if breakfast is skipped, high levels of MVPA could reduce the association of skipping breakfast on excess weight in young people.

One strength of the current study is that, to date, the moderator role of MVPA level in the relation between skipping breakfast and excess weight in young people has not been extensively studied. Thus, our study evaluated this association in a large sample of Spanish young people. Conversely, our research also had certain limitations. A causal relationship was not developed due to the cross-sectional nature of this study. Thus, it is not possible to conclude that engaging in MVPA (with or without skipping breakfast) promotes having excess weight or, contrariwise, that skipping breakfast causes decrease in the MVPA level (i.e., the reverse causality). Hence, despite the reason that a specific dependence direction between variables was discussed, several directions of these associations are also possible. In addition, the estimation of PA level would be more accurate with the use of accelerometers instead of questionnaires. However, Saint-Maurice et al.’s Equation [34] has shown a high correlation with accelerometry values (*r* = 0.63) and there were no statistically significant differences between the recorded and estimated MVPA. One difficulty found to analyze the evidence of the association between eating breakfast and health is the description of what a healthy breakfast includes, in relation to the type of food that it is composed of, the frequency of its consumption, or the energy content, aspects which were not analyzed in the present study [57]. Also, it has been pointed out that energy density (in breakfast) should be considered as a further element of breakfast intake [58], since it could be associated with cardiometabolic risk factors in young people with excess weight. Finally, fatness by a 4-compartment model, which is considered the gold standard for assessing body composition, was not measured. However, it is high-priced and involves specific tools to independently determine the body water, total bone mineral content, and body volume [59]. For all of these abovementioned reasons, caution is required to interpret our results.

In conclusion, our results showed that daily MVPA moderates the association between skipping breakfast and excess weight, meaning that PA of sufficient intensity seems to reduce the effect of skipping breakfast on young body weight status. This finding may be important for the establishment of public health policies that will help to the prevention of childhood obesity. Therefore, interventions planned to promote the sufficient practice of PA among children and adolescents could be crucial for maintaining a more appropriate weight status, especially in those who are skipping breakfast.

## Supplementary information

Below is the link to the electronic supplementary material.

Supplementary file1 (PDF 32 KB)

Supplementary file2 (DOCX 30 KB)

## Data Availability

The data generated and analyzed for the current study are available at reasonable request to the corresponding author.

## Abbreviations

BMI: Body mass index
KIDMED: Mediterranean Diet Quality Index for children and teenagers
MVPA: Moderate-to-vigorous physical activity (MVPA)
PA: Physical activity
PAQ-A: Physical Activity Questionnaire for Adolescents
PAQ-C: Physical Activity Questionnaire for Older Children
OR: Odds ratio
SES: Socioeconomic status
WHO: World Health Organization
