# Correction to: Skipping breakfast and excess weight among young people: the moderator role of moderate-to-vigorous physical activity

**DOI:** 10.1007/s00431-022-04527-3

**Published:** 2022-07-11

**Authors:** José Francisco López-Gil, Pedro Antonio Sánchez-Miguel, Miguel Ángel Tapia-Serrano, Antonio García-Hermoso

**Affiliations:** 1 Departamento de Expresión Plástica, Musical Y Dinámica, Facultad de Educación, Universidad de Murcia (UM), Región of Murcia, 30100 Murcia, Spain; 2 Health and Social Research Center, Universidad de Castilla-La Mancha (UCLM), 16071 Cuenca, Spain; 3 Department of Didactics of Musical, Plastic and Body Expression, Faculty of Teaching Training, University of Extremadura, Avenida Universidad, 10071 Cáceres, S/N Spain; 4 Hospital Universitario de Navarra (HUN), Universidad Pública de Navarra (UPNA), IdiSNA, 31008 NavarrabiomedPamplona, Navarra, Spain

## Correction to: European Journal of Pediatrics 10.1007/s00431-022-04503-x

The Fig. 1 in the original published version of the above article was incorrect. The corrected Figure is shown as follows:



The original article has been corrected.

