# Supplementary material for: Skipping breakfast and excess weight among young people: the moderator role of moderate-to-vigorous physical activity
Source: Eur J Pediatr. 2022 Jun 1;181(8):3195–204. doi: 10.1007/s00431-022-04503-x (PMC9352742; doi:10.1007/s00431-022-04503-x)
Supplement: Supplementary file 1 — Supplementary file1 (PDF 32 KB) [file 431_2022_4503_MOESM1_ESM.pdf]

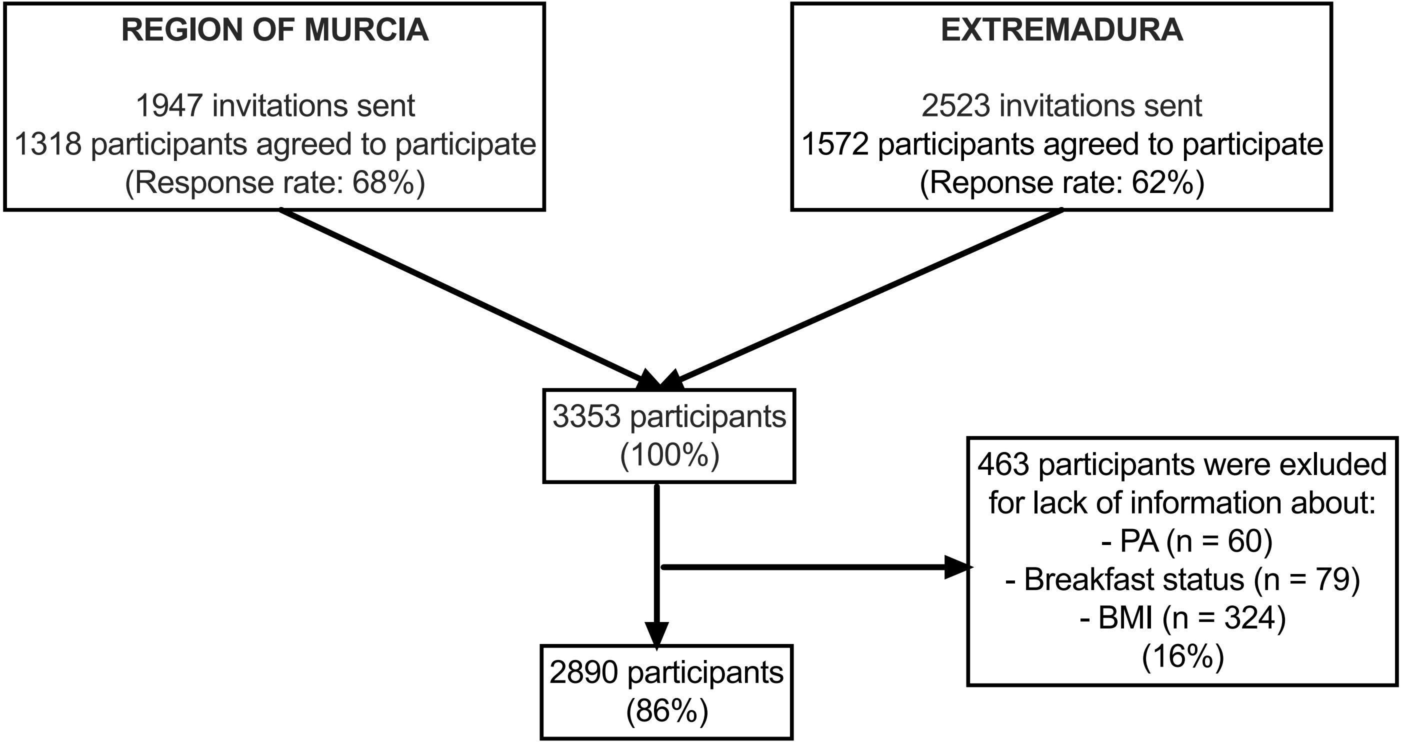
REGION OF MURCIA
1947 invitations sent
1318 participants agreed to participate
(Response rate: 68%)
EXTREMADURA
2523 invitations sent
1572 participants agreed to participate
(Reponse rate: 62%)
3353 participants
(100%)
463 participants were exluded
for lack of information about:
- PA (n = 60)
- Breakfast status (n = 79)
- BMI (n = 324)
(16%)
2890 participants
(86%)
