# Supplementary material for: Skipping breakfast and excess weight among young people: the moderator role of moderate-to-vigorous physical activity
Source: Eur J Pediatr. 2022 Jun 1;181(8):3195–204. doi: 10.1007/s00431-022-04503-x (PMC9352742; doi:10.1007/s00431-022-04503-x)
Supplement: Supplementary file 2 — Supplementary file2 (DOCX 30 KB) [file 431_2022_4503_MOESM2_ESM.docx]

**Table S1.** Characteristics and differences between study participants that were included or not in the final analysis.

| **Variables** | **Included** | | | **Missing** | | | ***p*** |
| --- | --- | --- | --- | --- | --- | --- | --- |
|  | **N** | **Mean / n** | **SD / %** | **N** | **Mean / n** | **SD / %** |  |
| Age (years) | 2890 | 12.4 | 2.7 | - | - | - | - |
| Avery income level (€) | 2890 | 20,527.3 | 2421.7 | - | - | - | - |
| Region of Murcia (%) | 2890 | 691 | 45 | - | - | - | - |
| Extremadura (%) | 2890 | 861 | 56 | - | - | - |  |
| Children (%) | 2890 | 660 | 43 | - | - | - | - |
| Adolescents (%) | 2890 | 892 | 58 | - | - | - |  |
| Weight (kg) | 2890 | 49.7 | 15.6 | 139 | 50.0 | 20.2 | 0.827 |
| Height (cm) | 2890 | 154.0 | 15.8 | 139 | 155.8 | 21.0 | 0.197 |
| BMI (z-score) ^a^ | 2890 | 0.7 | 1.2 | 139 | 0.8 | 1.2 | 0.337 |
| Excess weight status ^a^ (%, yes) | 2890 | 660 | 43 | 139 | 40 | 40 | 0.105 |
| Breakfast status (%, skipping) | 2890 | 143 | 9.2 | 384 | 26 | 8 | 0.126 |
| PAQ-C / PAQ-A (score) | 2890 | 1.7 | 0.1 | 403 | 1.7 | 0.2 | 0.170 |
| Daily MVPA mean (min) | 2890 | 6.4 | 3.2 | 403 | 6.3 | 3.6 | 0.563 |

BMI: body mass index. MVPA: moderate-to-vigorous physical activity. PAQ-A: Physical Activity Questionnaire for Adolescents; PAQ-C: Physical Activity Questionnaire for Older Children. ^a^ Excess weight established by the sum of participants with overweight or obesity according to the World Health Organization criteria^27,28^.
